# Antimicrobial resistance dynamics of *Listeria monocytogenes* in France: where we are and what we need?

**DOI:** 10.1016/j.lanepe.2024.100843

**Published:** 2024-01-16

**Authors:** Abdelaziz Ed-Dra

**Affiliations:** Laboratory of Engineering and Applied Technologies, Higher School of Technology, M’ghila Campus, Sultan Moulay Slimane University, Beni Mellal 23000, Morocco

Listeriosis, caused by *Listeria monocytogenes*, is a significant global foodborne illness with high mortality rate (20%–30%),^1^ especially affecting vulnerable populations.^2^ The primary approach for treating listeriosis involves the use of aminopenicillins alone or in combination with aminoglycosides.^2^ However, the persistent rise in antimicrobial resistance (AMR) has jeopardized current treatments. Therefore, global surveillance systems employing in-depth and accurate methods are crucial for comprehending the AMR mechanisms and, consequently, limiting the spread of antimicrobial-resistant strains, to safeguard the effectiveness of both current and future treatments.

In this issue of *The Lancet Regional Health—Europe*, Moura and colleagues^3^ have used massive sequencing data coupled with phenotypic AMR assessment of *L. monocytogenes* isolated from clinical (n = 2908) and food (n = 2431) samples in France and overseas territories. The findings indicate that there is no increase in AMR, and all isolates (100%) demonstrated susceptibility to ampicillin and amoxicillin, the first-line treatments for listeriosis. Additionally, the authors illustrated a high accuracy (>99%) of whole genome sequencing (WGS) in predicting AMR, along with the associated genetic elements such as transposons, prophages, or plasmids. Furthermore, the study revealed that while acquired resistance was infrequent (2.23%), it was more prevalent in food (3.74%) than clinical isolates (0.98%) (*p* < 0.0001). Notably, isolates exhibiting disinfectant or stress resistance traits (*bcrABC* and *emrC* genes encoding resistance to benzalkonium chloride) showed a higher prevalence of acquired resistance (20.20% vs. 7.20%) (*p* < 0.0001).

Interestingly, these findings provide evidence for the efficacy of the current listeriosis treatments. However, recent worldwide studies have indicated a rise in AMR, especially toward aminopenicillins,^4^, ^5^, ^6^ presenting another facet of a controversial picture. Significantly, the high accuracy observed in predicting AMR underscores the potential utility of WGS for routine surveillance of AMR in *L. monocytogenes*. Thus far, WGS has demonstrated high accuracy in predicting AMR in foodborne pathogens. Notably, Nguyen et al. have shown that MICs can be predicted with an average accuracy of 95%, within ±1 2-fold dilution step.^7^ Precisely, the high prevalence of acquired resistance in food isolates and in isolates with disinfectants or stress resistance traits, raises questions about the factors influencing the acquisition of antimicrobial resistance genes (ARGs), especially in food rather than clinical isolates. Beyond the use of antimicrobial agents in veterinary practices and animal-derived food production, the extensive use of disinfectants may contribute to the development of resistance even after the ban of antibiotics use.^8^ This emphasizes the potential for acquiring ARGs through co- and cross-resistance mechanisms influenced by disinfectants, environmental pollutants, or transfer by various vectors like phages.^9^^,^^10^

Despite the strengths highlighted by this study, some limitations should be addressed. First, the authors concluded that AMR of *L. monocytogenes* remained stable during the period of the study (2012–2019), with no detection of phenotypic and/or genotypic resistance toward the first line listeriosis treatments. This conclusion should be taken with caution since the bacterial adaptation to environmental stressors, including antimicrobials, is complex and depends on various factors (e.g. time of contact with stressors). Hence, a study covering isolates for a long period is more suitable and can guide us to make more precise decisions. Second, the prediction of ARGs from WGS requires more effort on developing accurate and reproductible machine-learning models to analyze the sequencing data in a large scale, in addition previous in-depth knowledge on genetic AMR is required to develop effective models, for example the low accuracy in predicting ciprofloxacin resistance can be due to the unknown mechanisms behind this resistance. Third, more isolates should be analyzed from overseas territories to provide accurate information about the possible transmission of AMR genotypes.

Given the imperative for more comprehensive global strategies to address the widespread dissemination of *L. monocytogenes* across countries and continents, influenced by extensive international travel and foods trading, there is a pressing need for effective measures. WGS has demonstrated its effectiveness in furnishing precise information regarding the main genotypes circulated in food and clinical samples, genotypic resistance exhibiting high concordance with phenotypic resistance, and the various mechanisms through which bacteria may acquire resistance genes, such as horizontal gene transfer, genomic mutations, co- and cross-resistance, among others. Furthermore, the growing number of available genome sequences worldwide necessitates the establishment of an integrated database for the international surveillance of *L. monocytogenes.* This database, based on WGS and complemented by epidemiological features of interest, seeks to unify, or link existing national networks into a more representative database. This database holds the potential to facilitate the sharing, analysis, and comparison of data from different countries, to trace the dissemination route of resistant *L. monocytogenes* strains, and to comprehend the mechanisms by which they develop and acquire AMR in an international context. Additionally, there is a need to establish interlaboratory tests to confirm critical resistances, develop quality controls for more accurate assays, and implement harmonized methods for reproducible experiments worldwide.

## Declaration of interests

The author has no conflicts of interest.
